# Crystal structure of (2*S*)-3-methyl-2-[(naphthalen-1-ylsulfon­yl)amino]­butanoic acid

**DOI:** 10.1107/S2056989015007057

**Published:** 2015-04-15

**Authors:** Muhammad Danish, Muhammad Nawaz Tahir, Nabila Jabeen, Muhammad Asam Raza

**Affiliations:** a Department of Chemistry, Institute of Natural Sciences, University of Gujrat, Gujrat 50700, Pakistan; b Department of Physics, University of Sargodha, Sargodha, Punjab, Pakistan

**Keywords:** crystal structure, catemer, naphthalen-1-ylsulfon­yl, l-valine, hydrogen bonding, π–π stacking inter­actions

## Abstract

The title compound, C_15_H_17_NO_4_S, was synthesized from l-valine and naphthalene-1-sulfonyl chloride. The hydrogen-bonded carb­oxy­lic acid groups form a catemer *C*(4) motif extending along [100]. The catemer structure is reinforced by a rather long N—H⋯O hydrogen bond, between the sulfamide N—H group and a carb­oxy­lic acid O atom [H⋯O = 2.52 (2) Å], and a C—H⋯O hydrogen bond.

## Related literature   

For related structures, see: Aguilar-Castro *et al.* (2004 ▸); Arshad *et al.* (2012 ▸); Mubashar-ur-Rehman *et al.* (2013 ▸).
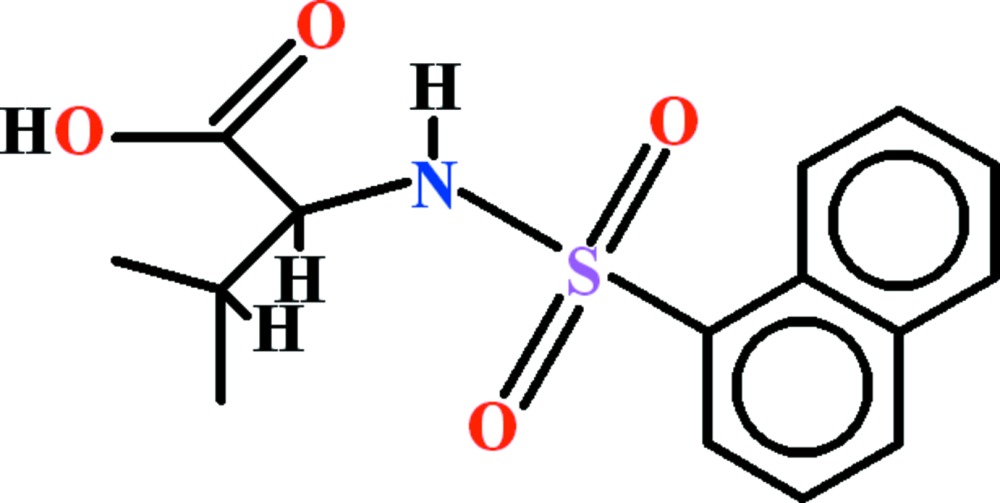


## Experimental   

### Crystal data  

C_15_H_17_NO_4_S
*M* *_r_* = 307.35
Orthorhombic, 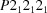

*a* = 5.5006 (3) Å
*b* = 13.7638 (8) Å
*c* = 20.2148 (14) Å
*V* = 1530.45 (16) Å^3^
*Z* = 4
Mo *K*α radiation
μ = 0.23 mm^−1^
*T* = 296 K
0.38 × 0.22 × 0.20 mm

### Data collection  

Bruker Kappa APEXII CCD diffractometer
Absorption correction: multi-scan (*SADABS*; Bruker, 2005 ▸) *T*_min_ = 0.920, *T*_max_ = 0.956
7706 measured reflections
3290 independent reflections
2692 reflections with *I* > 2σ(*I*)
*R*_int_ = 0.032

### Refinement  

*R*[*F* ^2^ > 2σ(*F* ^2^)] = 0.045
*wR*(*F* ^2^) = 0.095
*S* = 1.02
3290 reflections
198 parameters
H atoms treated by a mixture of independent and constrained refinement
Δρ_max_ = 0.22 e Å^−3^
Δρ_min_ = −0.24 e Å^−3^
Absolute structure: Flack *x* determined using 919 quotients [(*I* ^+^)-(*I* ^-^)]/[(*I* ^+^)+(*I* ^-^)] (Parsons *et al.*, 2013 ▸)
Absolute structure parameter: −0.05 (5)

### 

Data collection: *APEX2* (Bruker, 2007 ▸); cell refinement: *SAINT* (Bruker, 2007 ▸); data reduction: *SAINT*; program(s) used to solve structure: *SHELXS97* (Sheldrick, 2008 ▸); program(s) used to refine structure: *SHELXL97* (Sheldrick, 2008 ▸); molecular graphics: *ORTEP-3 for Windows* (Farrugia, 2012 ▸) and *PLATON* (Spek, 2009 ▸); software used to prepare material for publication: *WinGX* (Farrugia, 2012 ▸) and *PLATON*.

## Supplementary Material

Crystal structure: contains datablock(s) global, I. DOI: 10.1107/S2056989015007057/gk2628sup1.cif

Structure factors: contains datablock(s) I. DOI: 10.1107/S2056989015007057/gk2628Isup2.hkl

Supporting information file. DOI: 10.1107/S2056989015007057/gk2628Isup3.cml

**Fig. 1.**
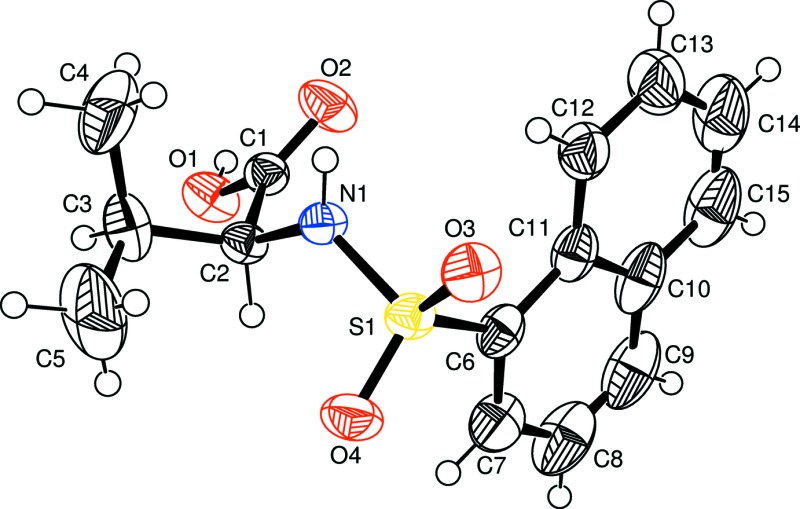
View of the asymmetric unit of title compound with the atom numbering scheme. The displacement ellipsoids are drawn at the 50% probability level. H-atoms are shown as small circles of arbitrary radii.

. DOI: 10.1107/S2056989015007057/gk2628fig1.tif
View of the asymmetric unit of title compound with the atom numbering scheme. The displacement ellipsoids are drawn at the 50% probability level. H-atoms are shown as small circles of arbitrary radii.

**Fig. 2.**
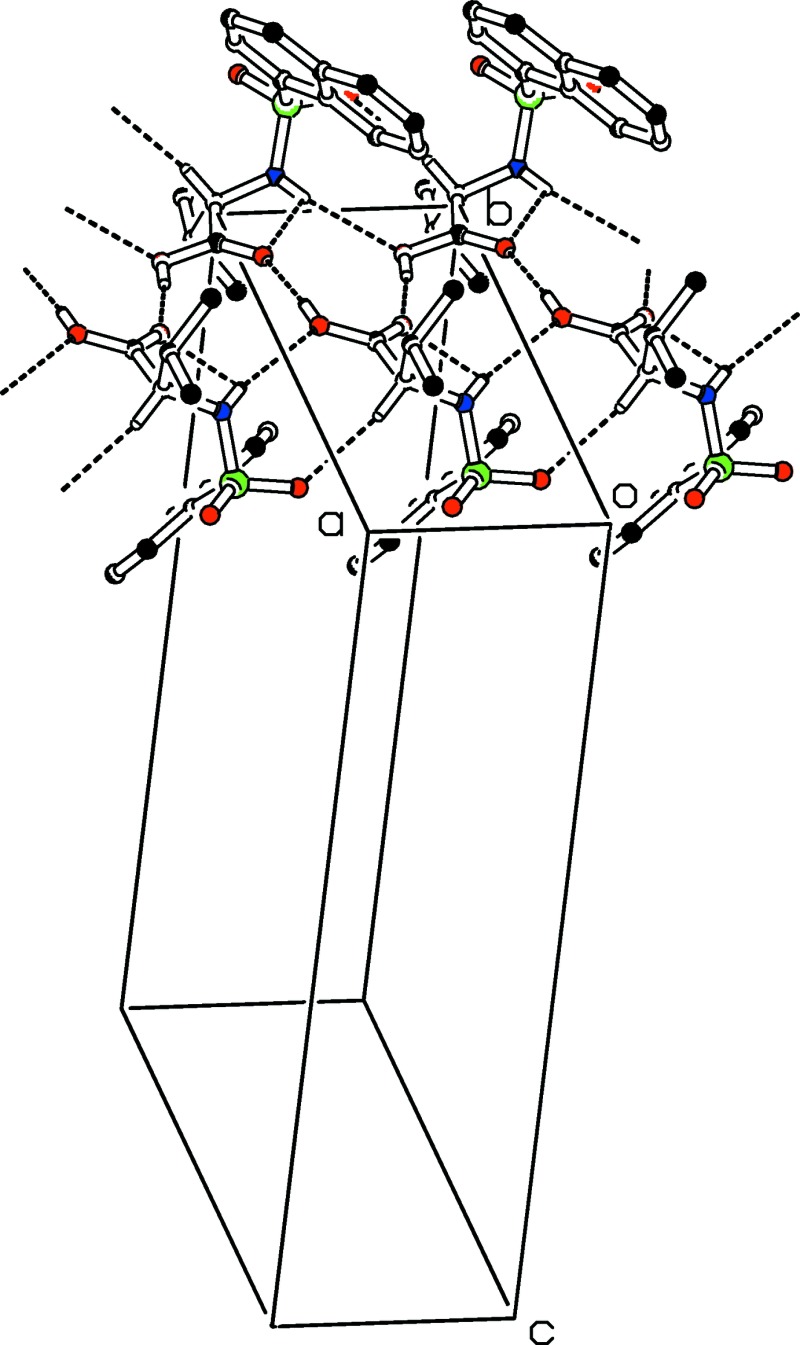
The partial packing (PLATON; Spek, 2009) which shows that molecules form one dimensional polymeric network with different hydrogen-bond ring motifs. H atoms not involved in hydrogen bonding are omitted for clarity.

PLATON . DOI: 10.1107/S2056989015007057/gk2628fig2.tif
The partial packing (*PLATON*; Spek, 2009) which shows that mol­ecules form one dimensional polymeric network with different hydrogen-bond ring motifs. H atoms not involved in hydrogen bonding are omitted for clarity.

CCDC reference: 1058549

Additional supporting information:  crystallographic information; 3D view; checkCIF report

## Figures and Tables

**Table 1:** Hydrogen-bond geometry (, )

*D*H*A*	*D*H	H*A*	*D* *A*	*D*H*A*
O1H1O2^i^	0.85(4)	1.86(4)	2.701(3)	173(4)
N1H1*A*O1^ii^	0.83(2)	2.52(2)	3.323(3)	166(3)
C2H2O3^iii^	0.98	2.38	3.348(4)	169

Symmetry codes: (i) 
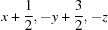
; (ii) 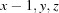; (iii) 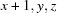.

## supplementary crystallographic information

### S1. Comment

The title compound (Fig. 1) was synthesized for complexation and other studies.

The crystal structures of *N*-(*p*-toluenesulfonyl)-*L*-valine (Aguilar-Castro *et al.*, 2004) and 2-benzenesulfonamido-3-methylbutyric acid (Arshad *et al.*, 2012) have been reported which contain the *L*-valine as common moiety as in (I). The crystal structure of 2- (naphthalene-1-sulfonamido)-3-phenylpropanoic acid (Mubashar-ur-Rehman *et al.*, 2013) has also been published which contains the naphthalene-1-sulfonamide group.

In (I), the aminoacetato moiety *A* (O1/O2/C1/C2/N1) of *L*-valine and naphthalene ring *B* (C6–C15) are planar with *r.m.s.* deviation of 0.0468 and 0.0163 Å, respectively. The dihedral angle between A/B is 69.26 (9)°. The sulfonyl group *C* (S1/O3/O4) is oriented at a dihedtal angle of 59.9 (1)° with the parent naphthalene ring. The H-atoms of carboxyl, amido and of substituted aminoacetato moiety are involved in H-bondings (Table 1, Fig. 2). There exist two types of ring motifs *R*_2_^2^(8) and *R*_3_^3^(11). The *R*_2_^2^(8) ring is formed due to C—H···O and N—H···O interactions. The *R*_3_^3^(11) ring is created due to O—H···O and N—H···O interactions in which three carboxyl groups are involved. The *R*_3_^3^(11) rings are connected successively along the *a*- axis, whereas, the *R*_2_^2^(8) rings are connected to *R*_3_^3^(11) rings alternatively, from opposite ends (Fig. 2).

### S2. Experimental

*L*-Valine (0.117 g, 1 mmol) and naphthalene-1-sulfonyl chloride (0.226 g, 1 mmol) were added to 30 ml of water. The reaction mixture was stirred at 323–328 K and pH of the reaction mixture was maintained at 8–9 by adding 1.0 *M* sodium bicarbonate solution. The heating was stopped when clear solution was obtained. After one hour 8 ml of 1.0 *M* HCl solution was added and white precipitate was formed. The precipitate was filtered and dried (yield: 70%; m.p. 421 K). White needles of the title compound were obtained after recrystallization from ethanol.

### S3. Refinement

The coordinates of H-atom of carboxyl and N–H group were freely refined. The other H atoms were positioned geometrically (C–H = 0.93—0.96 Å) and refined as riding with *U*_iso_(H) = *xU*_eq_(C, N, O), where *x* = 1.5 for hydroxy and *x* = 1.2 for all other H-atoms.

### Crystal data

**Table:**

C_15_H_17_NO_4_S	*D*_x_ = 1.334 Mg m^−^^3^
*M**_r_* = 307.35	Mo *K*α radiation, λ = 0.71073 Å
Orthorhombic, *P*2_1_2_1_2_1_	Cell parameters from 2692 reflections
*a* = 5.5006 (3) Å	θ = 3.1–27.0°
*b* = 13.7638 (8) Å	µ = 0.23 mm^−^^1^
*c* = 20.2148 (14) Å	*T* = 296 K
*V* = 1530.45 (16) Å^3^	Needle, colorless
*Z* = 4	0.38 × 0.22 × 0.20 mm
*F*(000) = 648	

### Data collection

**Table:**

Bruker Kappa APEXII CCD diffractometer	3290 independent reflections
Radiation source: fine-focus sealed tube	2692 reflections with *I* > 2σ(*I*)
Graphite monochromator	*R*_int_ = 0.032
Detector resolution: 7.80 pixels mm^-1^	θ_max_ = 27.0°, θ_min_ = 3.1°
ω scans	*h* = −6→7
Absorption correction: multi-scan (*SADABS*; Bruker, 2005)	*k* = −10→17
*T*_min_ = 0.920, *T*_max_ = 0.956	*l* = −21→25
7706 measured reflections	

### Refinement

**Table:**

Refinement on *F*^2^	Secondary atom site location: difference Fourier map
Least-squares matrix: full	Hydrogen site location: inferred from neighbouring sites
*R*[*F*^2^ > 2σ(*F*^2^)] = 0.045	H atoms treated by a mixture of independent and constrained refinement
*wR*(*F*^2^) = 0.095	*w* = 1/[σ^2^(*F*_o_^2^) + (0.0423*P*)^2^ + 0.0587*P*] where *P* = (*F*_o_^2^ + 2*F*_c_^2^)/3
*S* = 1.02	(Δ/σ)_max_ < 0.001
3290 reflections	Δρ_max_ = 0.22 e Å^−^^3^
198 parameters	Δρ_min_ = −0.24 e Å^−^^3^
0 restraints	Absolute structure: Flack *x* determined using 919 quotients [(*I*^+^)-(*I*^-^)]/[(*I*^+^)+(*I*^-^)] (Parsons *et al.*, 2013)
Primary atom site location: structure-invariant direct methods	Absolute structure parameter: −0.05 (5)

### Special details

**Table:**

Geometry. All esds (except the esd in the dihedral angle between two l.s. planes) are estimated using the full covariance matrix. The cell esds are taken into account individually in the estimation of esds in distances, angles and torsion angles; correlations between esds in cell parameters are only used when they are defined by crystal symmetry. An approximate (isotropic) treatment of cell esds is used for estimating esds involving l.s. planes.
Refinement. Refinement of *F*^2^ against ALL reflections. The weighted *R*-factor *wR* and goodness of fit *S* are based on *F*^2^, conventional *R*-factors *R* are based on *F*, with *F* set to zero for negative *F*^2^. The threshold expression of *F*^2^ > σ(*F*^2^) is used only for calculating *R*-factors(gt) *etc*. and is not relevant to the choice of reflections for refinement. *R*-factors based on *F*^2^ are statistically about twice as large as those based on *F*, and *R*-factors based on ALL data will be even larger.

### Fractional atomic coordinates and isotropic or equivalent isotropic displacement parameters (Å^2^)

**Table:**

	*x*	*y*	*z*	*U*_iso_*/*U*_eq_	
S1	0.22778 (13)	0.44851 (5)	0.11598 (4)	0.0360 (2)	
O1	0.8107 (4)	0.63127 (18)	−0.00511 (13)	0.0473 (6)	
H1	0.836 (7)	0.692 (3)	−0.008 (2)	0.071*	
O2	0.4319 (5)	0.67953 (17)	0.01812 (14)	0.0549 (7)	
O3	−0.0306 (4)	0.43808 (16)	0.11995 (12)	0.0463 (6)	
O4	0.3786 (4)	0.36514 (16)	0.12075 (12)	0.0509 (6)	
N1	0.2790 (4)	0.49709 (18)	0.04429 (12)	0.0328 (6)	
H1A	0.180 (5)	0.539 (2)	0.0325 (16)	0.039*	
C1	0.5806 (6)	0.6161 (2)	0.01096 (15)	0.0349 (7)	
C2	0.5237 (5)	0.5091 (2)	0.01805 (15)	0.0329 (7)	
H2	0.6386	0.4809	0.0497	0.039*	
C3	0.5555 (6)	0.4565 (3)	−0.04864 (19)	0.0510 (9)	
H3	0.7206	0.4699	−0.0644	0.061*	
C4	0.3806 (8)	0.4953 (4)	−0.0997 (2)	0.0839 (15)	
H4A	0.4062	0.4623	−0.1409	0.126*	
H4B	0.4077	0.5637	−0.1056	0.126*	
H4C	0.2168	0.4847	−0.0850	0.126*	
C5	0.5323 (12)	0.3476 (3)	−0.0401 (3)	0.105 (2)	
H5A	0.6424	0.3261	−0.0063	0.158*	
H5B	0.5711	0.3159	−0.0811	0.158*	
H5C	0.3687	0.3318	−0.0276	0.158*	
C6	0.3219 (5)	0.5300 (3)	0.17904 (16)	0.0410 (8)	
C7	0.5143 (6)	0.5024 (3)	0.21720 (19)	0.0572 (10)	
H7	0.5961	0.4449	0.2077	0.069*	
C8	0.5895 (8)	0.5597 (4)	0.2704 (2)	0.0786 (15)	
H8	0.7216	0.5402	0.2959	0.094*	
C9	0.4737 (8)	0.6422 (4)	0.2851 (2)	0.0762 (15)	
H9	0.5258	0.6789	0.3211	0.091*	
C10	0.2755 (8)	0.6745 (3)	0.24761 (19)	0.0611 (11)	
C11	0.1958 (6)	0.6187 (2)	0.19223 (17)	0.0447 (8)	
C12	−0.0009 (7)	0.6539 (3)	0.1546 (2)	0.0542 (10)	
H12	−0.0554	0.6185	0.1183	0.065*	
C13	−0.1127 (9)	0.7393 (3)	0.1708 (2)	0.0769 (14)	
H13	−0.2395	0.7624	0.1447	0.092*	
C14	−0.0376 (13)	0.7922 (3)	0.2262 (3)	0.0939 (19)	
H14	−0.1174	0.8495	0.2375	0.113*	
C15	0.1496 (11)	0.7608 (4)	0.2635 (2)	0.0829 (17)	
H15	0.1967	0.7965	0.3003	0.099*	

### Atomic displacement parameters (Å^2^)

**Table:**

	*U*^11^	*U*^22^	*U*^33^	*U*^12^	*U*^13^	*U*^23^
S1	0.0350 (4)	0.0333 (4)	0.0397 (4)	−0.0026 (4)	0.0009 (3)	0.0062 (4)
O1	0.0410 (14)	0.0304 (12)	0.0705 (17)	−0.0076 (11)	0.0045 (11)	0.0069 (13)
O2	0.0536 (15)	0.0311 (12)	0.0799 (19)	0.0074 (13)	0.0100 (13)	0.0071 (13)
O3	0.0354 (11)	0.0510 (13)	0.0525 (14)	−0.0103 (10)	0.0034 (10)	0.0064 (13)
O4	0.0541 (13)	0.0363 (12)	0.0622 (16)	0.0081 (11)	0.0020 (13)	0.0133 (13)
N1	0.0310 (13)	0.0309 (13)	0.0364 (14)	0.0026 (12)	0.0001 (11)	0.0053 (11)
C1	0.0378 (17)	0.0315 (17)	0.0354 (17)	0.0015 (15)	−0.0021 (14)	0.0008 (14)
C2	0.0307 (16)	0.0291 (16)	0.0390 (18)	−0.0006 (13)	0.0007 (13)	0.0041 (15)
C3	0.0468 (19)	0.044 (2)	0.062 (2)	−0.0076 (18)	0.0161 (17)	−0.0155 (19)
C4	0.087 (3)	0.120 (4)	0.045 (3)	−0.014 (3)	−0.002 (2)	−0.024 (3)
C5	0.152 (5)	0.045 (3)	0.119 (5)	−0.008 (3)	0.042 (4)	−0.031 (3)
C6	0.0362 (17)	0.052 (2)	0.0351 (18)	−0.0101 (15)	0.0041 (13)	0.0047 (15)
C7	0.041 (2)	0.081 (3)	0.050 (2)	−0.005 (2)	−0.0005 (16)	0.004 (2)
C8	0.054 (2)	0.135 (5)	0.048 (3)	−0.023 (3)	−0.010 (2)	0.004 (3)
C9	0.077 (3)	0.111 (4)	0.040 (2)	−0.040 (3)	−0.001 (2)	−0.011 (3)
C10	0.076 (3)	0.064 (2)	0.044 (2)	−0.030 (3)	0.018 (2)	−0.0049 (19)
C11	0.053 (2)	0.0432 (19)	0.0378 (18)	−0.0135 (18)	0.0101 (16)	−0.0006 (16)
C12	0.068 (2)	0.047 (2)	0.047 (2)	0.005 (2)	0.0091 (19)	−0.0024 (19)
C13	0.102 (3)	0.058 (3)	0.071 (3)	0.023 (3)	0.023 (3)	0.004 (2)
C14	0.157 (6)	0.045 (3)	0.079 (4)	0.011 (3)	0.047 (4)	−0.007 (3)
C15	0.136 (5)	0.056 (3)	0.056 (3)	−0.027 (3)	0.026 (3)	−0.020 (2)

### Geometric parameters (Å, º)

**Table:**

S1—O4	1.419 (2)	C5—H5C	0.9600
S1—O3	1.431 (2)	C6—C7	1.364 (4)
S1—N1	1.621 (3)	C6—C11	1.430 (5)
S1—C6	1.775 (3)	C7—C8	1.397 (6)
O1—C1	1.323 (4)	C7—H7	0.9300
O1—H1	0.85 (4)	C8—C9	1.335 (7)
O2—C1	1.205 (4)	C8—H8	0.9300
N1—C2	1.456 (4)	C9—C10	1.401 (6)
N1—H1A	0.83 (2)	C9—H9	0.9300
C1—C2	1.513 (4)	C10—C15	1.412 (6)
C2—C3	1.540 (5)	C10—C11	1.426 (5)
C2—H2	0.9800	C11—C12	1.408 (5)
C3—C4	1.508 (6)	C12—C13	1.366 (5)
C3—C5	1.514 (6)	C12—H12	0.9300
C3—H3	0.9800	C13—C14	1.400 (7)
C4—H4A	0.9600	C13—H13	0.9300
C4—H4B	0.9600	C14—C15	1.347 (7)
C4—H4C	0.9600	C14—H14	0.9300
C5—H5A	0.9600	C15—H15	0.9300
C5—H5B	0.9600		
			
O4—S1—O3	119.71 (14)	C3—C5—H5C	109.5
O4—S1—N1	107.01 (14)	H5A—C5—H5C	109.5
O3—S1—N1	105.33 (14)	H5B—C5—H5C	109.5
O4—S1—C6	106.96 (16)	C7—C6—C11	120.6 (3)
O3—S1—C6	108.23 (15)	C7—C6—S1	117.2 (3)
N1—S1—C6	109.32 (14)	C11—C6—S1	122.2 (2)
C1—O1—H1	109 (3)	C6—C7—C8	120.5 (4)
C2—N1—S1	122.2 (2)	C6—C7—H7	119.7
C2—N1—H1A	115 (2)	C8—C7—H7	119.7
S1—N1—H1A	115 (2)	C9—C8—C7	120.7 (4)
O2—C1—O1	124.3 (3)	C9—C8—H8	119.7
O2—C1—C2	123.6 (3)	C7—C8—H8	119.7
O1—C1—C2	112.0 (3)	C8—C9—C10	121.4 (4)
N1—C2—C1	109.6 (2)	C8—C9—H9	119.3
N1—C2—C3	111.8 (2)	C10—C9—H9	119.3
C1—C2—C3	110.5 (3)	C9—C10—C15	121.7 (5)
N1—C2—H2	108.3	C9—C10—C11	119.6 (4)
C1—C2—H2	108.3	C15—C10—C11	118.7 (4)
C3—C2—H2	108.3	C12—C11—C10	118.4 (4)
C4—C3—C5	112.0 (4)	C12—C11—C6	124.5 (3)
C4—C3—C2	111.1 (3)	C10—C11—C6	117.2 (3)
C5—C3—C2	110.8 (3)	C13—C12—C11	120.8 (4)
C4—C3—H3	107.5	C13—C12—H12	119.6
C5—C3—H3	107.5	C11—C12—H12	119.6
C2—C3—H3	107.5	C12—C13—C14	120.4 (5)
C3—C4—H4A	109.5	C12—C13—H13	119.8
C3—C4—H4B	109.5	C14—C13—H13	119.8
H4A—C4—H4B	109.5	C15—C14—C13	120.4 (5)
C3—C4—H4C	109.5	C15—C14—H14	119.8
H4A—C4—H4C	109.5	C13—C14—H14	119.8
H4B—C4—H4C	109.5	C14—C15—C10	121.2 (5)
C3—C5—H5A	109.5	C14—C15—H15	119.4
C3—C5—H5B	109.5	C10—C15—H15	119.4
H5A—C5—H5B	109.5		
			
O4—S1—N1—C2	−44.2 (3)	S1—C6—C7—C8	−176.0 (3)
O3—S1—N1—C2	−172.6 (2)	C6—C7—C8—C9	0.4 (6)
C6—S1—N1—C2	71.3 (3)	C7—C8—C9—C10	−0.6 (7)
S1—N1—C2—C1	−115.5 (3)	C8—C9—C10—C15	178.9 (4)
S1—N1—C2—C3	121.6 (3)	C8—C9—C10—C11	−0.4 (6)
O2—C1—C2—N1	−8.8 (4)	C9—C10—C11—C12	−178.8 (3)
O1—C1—C2—N1	172.4 (3)	C15—C10—C11—C12	1.9 (5)
O2—C1—C2—C3	114.9 (3)	C9—C10—C11—C6	1.6 (5)
O1—C1—C2—C3	−64.0 (3)	C15—C10—C11—C6	−177.8 (3)
N1—C2—C3—C4	60.0 (4)	C7—C6—C11—C12	178.6 (3)
C1—C2—C3—C4	−62.4 (4)	S1—C6—C11—C12	−4.7 (4)
N1—C2—C3—C5	−65.3 (4)	C7—C6—C11—C10	−1.8 (5)
C1—C2—C3—C5	172.3 (4)	S1—C6—C11—C10	174.9 (2)
O4—S1—C6—C7	2.0 (3)	C10—C11—C12—C13	0.0 (5)
O3—S1—C6—C7	132.3 (3)	C6—C11—C12—C13	179.6 (4)
N1—S1—C6—C7	−113.5 (3)	C11—C12—C13—C14	−1.7 (6)
O4—S1—C6—C11	−174.8 (2)	C12—C13—C14—C15	1.6 (8)
O3—S1—C6—C11	−44.5 (3)	C13—C14—C15—C10	0.4 (8)
N1—S1—C6—C11	69.7 (3)	C9—C10—C15—C14	178.6 (4)
C11—C6—C7—C8	0.9 (5)	C11—C10—C15—C14	−2.1 (7)

### Hydrogen-bond geometry (Å, º)

**Table:**

*D*—H···*A*	*D*—H	H···*A*	*D*···*A*	*D*—H···*A*
O1—H1···O2^i^	0.85 (4)	1.86 (4)	2.701 (3)	173 (4)
N1—H1*A*···O1^ii^	0.83 (2)	2.52 (2)	3.323 (3)	166 (3)
C2—H2···O3^iii^	0.98	2.38	3.348 (4)	169

Symmetry codes: (i) *x*+1/2, −*y*+3/2, −*z*; (ii) *x*−1, *y*, *z*; (iii) *x*+1, *y*, *z*.
