# Supplementary figures and images for: Crystal structure of (2S)-3-methyl-2-[(naphthalen-1-ylsulfon­yl)amino]­butanoic acid
Source: Acta Crystallogr E Crystallogr Commun. 2015 Apr 15;71(Pt 5):o308. doi: 10.1107/S2056989015007057 (PMC4420138; doi:10.1107/S2056989015007057)

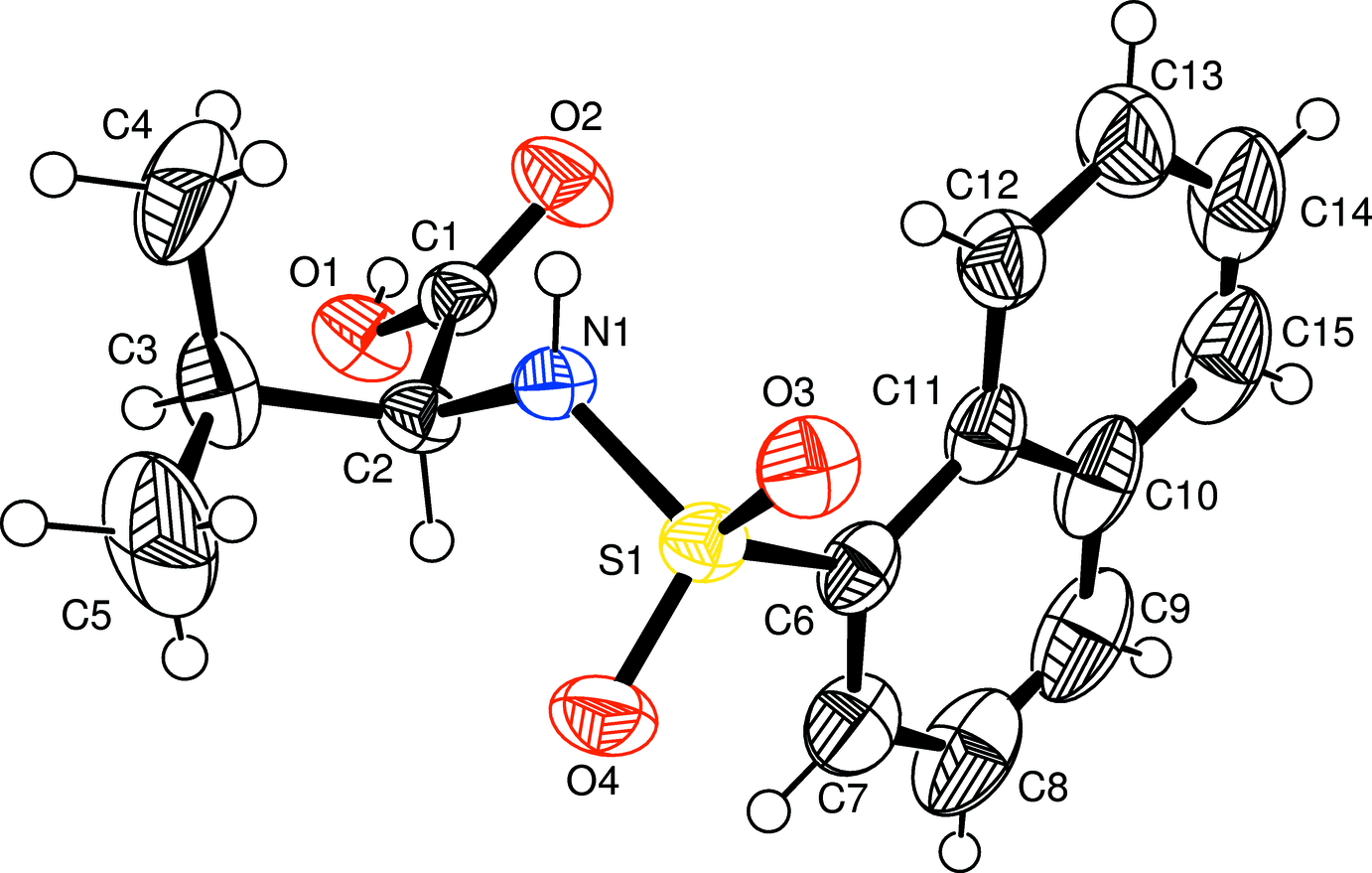

Supplement: Supplementary file 4 [file e-71-0o308-fig1.tif]

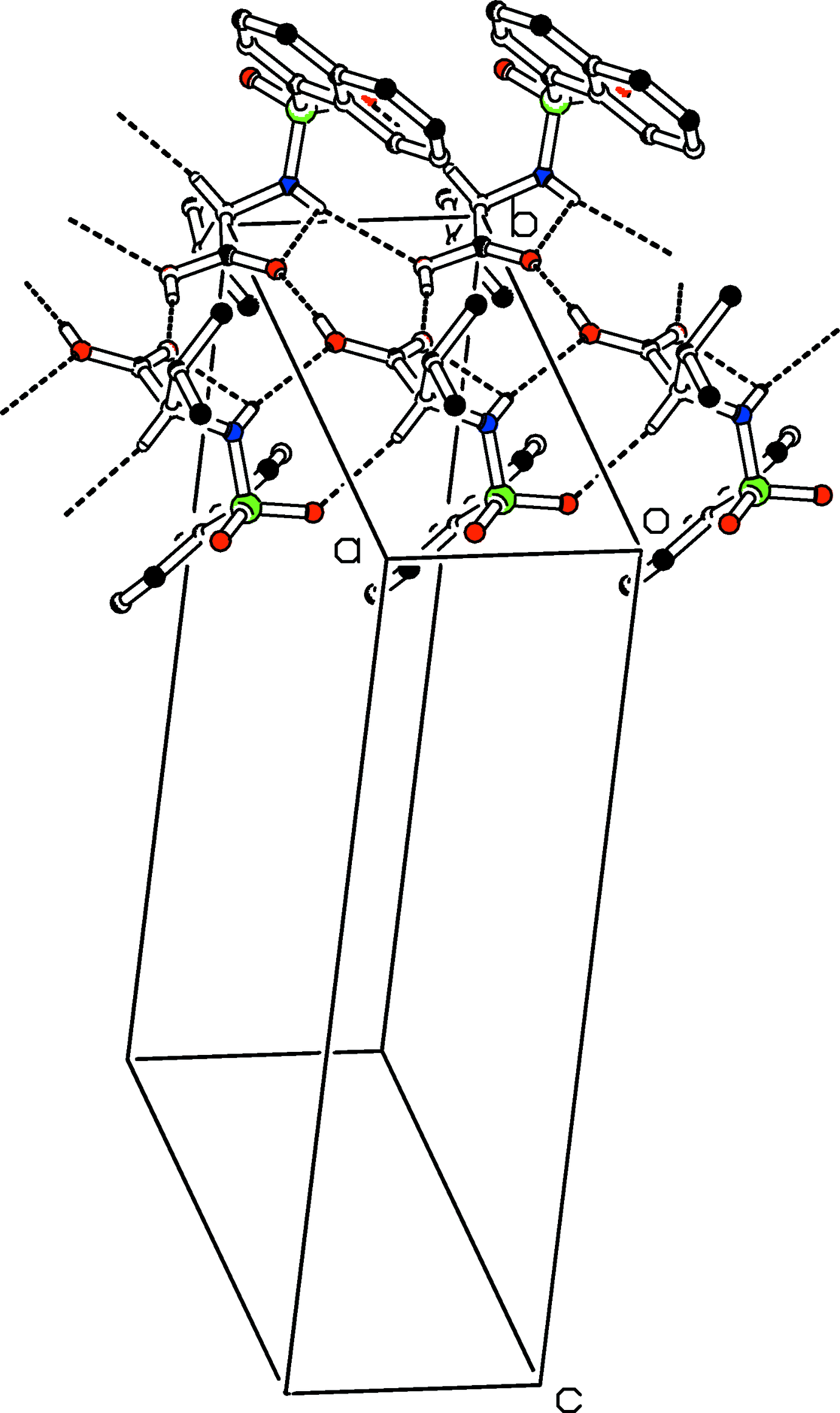

Supplement: Supplementary file 5 [file e-71-0o308-fig2.tif]
